# Absorptive stripping voltammetry for cannabis detection

**DOI:** 10.1186/s13065-015-0117-0

**Published:** 2015-07-01

**Authors:** Rita Nissim, Richard G Compton

**Affiliations:** Department of Chemistry, Physical & Theoretical Chemistry Laboratory, Oxford University, South Parks Road, Oxford, OX1 3QZ UK

**Keywords:** Absorptive stripping voltammetry, Δ^9^-tetrahydrocannabinol, Cannabis detection, THC detection, Carbon paste electrode, Liquid-liquid interfaces

## Abstract

**Background:**

Given that Δ^9^-tetrahydrocannabinol, the active constituent of cannabis, has been shown to greatly reduce driving ability, thus being linked to many drug driving accidents, its reliable detection is of great importance.

**Results:**

An optimised carbon paste electrode, fabricated from graphite powder and mineral oil, is utilised for the sensitive detection of Δ^9^-tetrahydrocannabinol (THC) in both aqueous solutions of pH 10.0 and in synthetic saliva solutions. “Absorptive Stripping Voltammetry” is exploited to that effect and the paste is used to pre-concentrate the carbon paste electrode with the target molecule. Practical limits of detection of 0.50 μM and 0.10 μM are determined for THC in stationary and stirred aqueous borate buffer solutions, respectively. Theoretical limits of detection are also calculated; values of 0.48 nM and 0.41 nM are determined for stationary and stirred THC aqueous borate buffer solutions, respectively. THC concentrations as low as 0.50 μM are detected in synthetic saliva solutions. The sensitivity of the sensor was 0.12 μA μM^−1^, 0.84 μA μM^−1^ and 0.067 μA μM^−1^ for the stationary buffer, the stirred buffer and the saliva matrix, respectively.

**Conclusions:**

“Absorptive Stripping Voltammetry” can be reliably applied to the detection of Δ^9^-tetrahydrocannabinol, after suitable optimisation of the assay. Usefully low practical limits of detection can be achieved.

## Background

Δ^9^-tetrahydrocannabinol (THC), the active constituent of cannabis, is known to reduce psychomotor function and cognition thus greatly impairing driving ability [1, 2]. As would be expected, reports indicate that the degree of impairment is related to the amount of THC present in the body; as low as 0.95 μM of THC in blood has been found to cause equivalent driving impairment to an alcohol blood concentration of 11.5 mM, the legal limit in most European countries [1]. At the same time, studies have also shown that cannabis is not only one of the most commonly used illegal drugs [3, 4] but that it is also associated with many drug driving motor vehicle accidents [5]. There is hence a great need for both accurate and low-concentration detection as well as the development of methodologies suitable for road-side detection.

Cannabinoids are usually detected using gas chromatography [6–8] or high-performance liquid chromatography, coupled to electrochemical detection [9–11]. However, given that electrochemical sensors are known for their high reliability and speed of response, their low cost and their compatibility for miniaturisation [12], they offer an attractive portable alternative [13–15]. Such sensors often rely on the indirect detection of THC, with sensing molecules including Gibb’s Reagent [13] and 4-aminophenol [14]. This is because the direct oxidation of the hydroxyl group leads to the formation of radicals and radical cations which foul the electrode surface [16–18]. This can significantly decrease the reproducibility of the obtained responses if the sample is other than very dilute.

As previous studies have shown, carbon paste electrodes may, under open circuit conditions, be “loaded” with an analyte of interest by being immersed in a solution containing that analyte [19–23]. The electrode can then be transferred into a blank solution, containing only buffer and supporting electrolyte, in which the appropriate measurement can be carried out. It is likely that the electrochemical reaction occurs at the triple phase boundary that exists at the carbon-oil (binder)-water triple interface [22], as the analyte diffuses out of the paste. By using the bulk-paste of an optimised carbon paste electrode to pre-concentrate THC and subsequently “stripping” that target, the effect of fouling in the less dilute samples can be overcome [20]. The pre-concentration of the target also ensures low limits of detection. This electroanalytical approach has been termed “absorptive stripping voltammetry”, with concentration in the electrode bulk [20], as opposed to “adsorptive stripping voltammetry” where the target is allowed to adsorb on the electrode surface.

The aim of this paper is to apply absorptive striping voltammetry to the detection of low THC concentrations in both aqueous solutions and in synthetic saliva, where the detection of THC will rely on the direct oxidation of the molecule on an optimised carbon paste electrode. The value of this approach has previously been indicated through work on the detection of different target molecules such as superoxide [19], phenol [20] and vitamin K1 [21]. Here, the oxidation signal of THC, observed at peak potentials of ca. +0.36 V [vs. saturated calomel electrode (SCE)], was used as the detection signal, with the carbon paste being fabricated from graphite and mineral oil.

## Experimental

### Chemical reagents

All reagents were purchased from Aldrich (Gillingham, U.K.), at the highest grade available, and were used as received, without any further purification. These were Δ^9^-Tetrahydrocannabinol (THC), dioctyl phthalate, mineral oil, graphite powder (particle diameter < 20 μm), synthetic saliva, sodium hydroxide (NaOH), sodium tetraborate (Na_2_B_4_O_7_) and potassium chloride (KCl). All aqueous solutions were prepared daily, at 298 K, using deionised water of resistivity of no less than 18.2 MΩ cm (25 °C, Millipore UHQ, Vivendi, U.K.) as the solvent and KCl (0.1 M) as the supporting electrolyte. They were made at pH = 10.0, achievable through the use of appropriate NaOH/ Na_2_B_4_O_7_ borate buffers (BBS solutions) and confirmed using a Hannah pH 213 pH meter. Where experiments required the absence of oxygen [24], solutions were deoxygenated using oxygen-free nitrogen (N_2_, BOC, Guildford, U.K.), in an air-tight environment, for at least 30 min. The measurements themselves were carried out under a light N_2_ flow.

### Equipment and experimental set-up

Square wave voltammetric measurements were recorded using a computer controlled Autolab potentiostat (PGSTAT 101, EcoChemie, Utrecht, Netherlands), in a home-built Faraday cage. A standard three-electrode configuration was used, with a carbon paste electrode (1.97 mm radius, 1.00 mm depth, made in-house) acting as the working electrode. A platinum wire (99.99 % GoodFellow, Cambridge, U.K.) was utilised as the counter electrode and a Saturated Calomel reference electrode (SCE, BAS Inc, Japan) completed the assembly. All experiments were carried out in a thermostated water bath, at a temperature of 25 ± 0.1 °C.

### Fabrication and characterisation of the carbon paste working electrodes

The carbon paste electrode holder was made from a copper rod of radius of 1.97 mm running through a Teflon rod (for electrical contact), leaving a 1.00 mm deep cavity at the edge. Two pastes were used, fabricated by mixing graphite powder with each liquid binder (dioctyl phthalate and mineral oil). The graphite/dioctyl phthalate paste, fabricated by mixing 1.4 mL dioctyl phthalate and 4.26 g graphite powder, has previously been characterised in aqueous potassium ferricyanide [19]; the same ratio of pasting liquid to carbon powder was thus used to make the graphite/mineral oil paste, unless otherwise stated.

### Working electrode surface preparation

The surface of the carbon paste electrodes was renewed between each scan by cleaning the holder and packing fresh paste. The errors in the calibration curves relate to separate electrode preparations.

## Results and discussion

The application of absorptive stripping voltammetry to the detection of THC is here discussed. Given previous work [20] on the detection of phenols using this approach, a pre-concentration time of 3 min was deemed adequate to equilibrate the paste with THC. An optimised paste composition was used, where graphite powder and mineral oil were used for its fabrication; this will be further discussed in Section 3.1. Practically useful limits of detection were thus achieved, with the oxidation signal of THC, observed at peak potentials of ca. +0.35 V (vs. SCE), being used as the detection signal.

### Selecting a carbon paste electrode

The electrochemical oxidation of THC was first investigated using square wave voltammetry. Two carbon paste electrodes, fabricated by mixing graphite powder with dioctyl phthalate or mineral oil as described in Section 2.3, were used aiming to select the carbon paste electrode that would ensure the highest THC uptake.

**Scheme 1:**
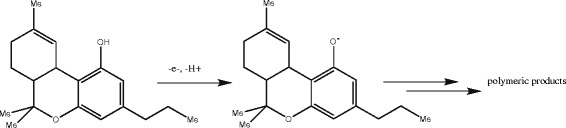
The oxidation of Δ^9^-tetrahydrocannabinol (THC). THC has been assumed to behave as a typical phenol [17, 25, 26]

**Fig. 1:**
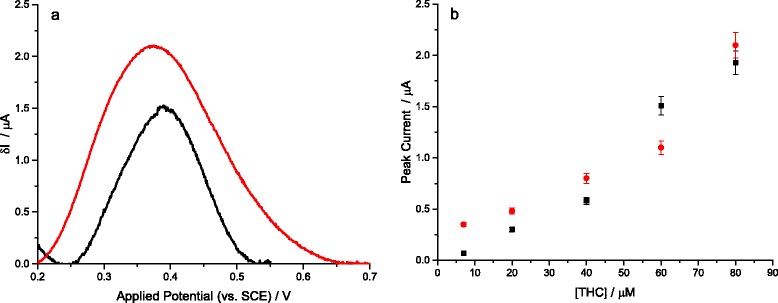
**a** Square wave voltammograms (frequency: 100 Hz, amplitude: 40 mV, step potential: 1 mV) for the oxidation of THC, seen at +0.37 V (vs. SCE), on the graphite/dioctyl phthalate paste electrode (black line) and the graphite/mineral oil paste electrode (red line). The measurement was obtained in a deoxygenated BBS (0.1 M KCl, pH = 10.0, 298 K), after immersing the electrode in identical stationary solutions that contained 80 μM THC, for 3 min. **b** Comparison of the peak currents obtained with the two carbon paste electrodes (black squares: graphite/dioctyl phthalate paste, red circles: graphite/mineral oil paste). The measurements were obtained in a deoxygenated BBS (0.1 M KCl, pH = 10.0, 298 K), after immersing the electrode in identical *stationary* solutions that contained 7.0 – 80 μM THC, for 3 min. The errors relate to separate electrode preparations

Each carbon paste electrode was immersed for 3 min, under open circuit conditions, in a deoxygenated aqueous borate buffer solution of pH 10.0 that contained 7.0 – 80 μM THC and 0.1 M KCl as the supporting electrolyte. Each paste electrode was then transferred to a deoxygenated aqueous borate buffer solution of pH 10.0, which only contained 0.1 M KCl. Oxidative square wave voltammetric scans were then run, between +0.25 V and + 0.52 V (vs. SCE) for the graphite/dioctyl phthalate paste electrode and between +0.20 V and +0.60 V (vs. SCE) in the case of the graphite/mineral oil paste electrode. The frequency and step potential used were 100 Hz and 1 mV, respectively, while the amplitude was set to 40 mV. Peaks due to the oxidation of THC, as shown in Scheme 1 [17, 25, 26], were seen at peak potentials of +0.39 V and +0.37 V (vs. SCE) on the graphite/dioctyl phthalate and the graphite/mineral oil pastes respectively; typical responses are depicted in Fig. 1. Scheme 1 assumes that THC behaves like a typical phenol [17]; to the best of the author’s knowledge there is no literature reporting detection of further THC oxidation products.

As can be seen in Fig. 1a, b, the peak current observed with the graphite/mineral oil paste was higher than that observed with the graphite/dioctyl phthalate paste, this reflecting the greater ability of mineral oil to accumulate THC. As depicted in Fig. 1b, the current increased with increasing analyte concentration, reflecting the increasing amount of THC transferring into the paste. Only the graphite/mineral oil paste electrode was used for further experiments to achieve a lower THC limit of detection.

### Obtaining analytical limits of detection for THC

#### Detecting THC in a stationary vs. a stirred aqueous solution

Focusing on lower THC concentrations, the graphite/mineral oil paste was tested in aqueous THC solutions of concentrations of 0.50 – 16 μM. Analogous experiments as in the Section above were thus carried out, where the pre-concentration time was increased to 5 min and where the amount of mineral oil used was doubled; the amount of graphite powder was kept constant. This was done to improve the uptake of THC and hence the sensitivity of the sensor.

The graphite/mineral oil carbon paste electrode was immersed in a stationary deoxygenated aqueous borate buffer solution of pH 10.0 that contained a known amount of THC (between 0.50 and 16 μM) and 0.1 M KCl as the supporting electrolyte. This was again done under open circuit conditions. Oxidative square wave voltammetric scans were then run in a deoxygenated aqueous borate buffer solution of pH 10.0, which only contained 0.1 M KCl, in the same potential range (between +0.20 V and +0.60 V [vs. SCE]) and using the same values of 100 Hz, 1 mV and 40 mV for the frequency, step potential and amplitude, respectively, as in the Section above. The same procedure was then repeated, stirring the source THC solutions to lower the limit of detection of the sensor.

**Fig. 2:**
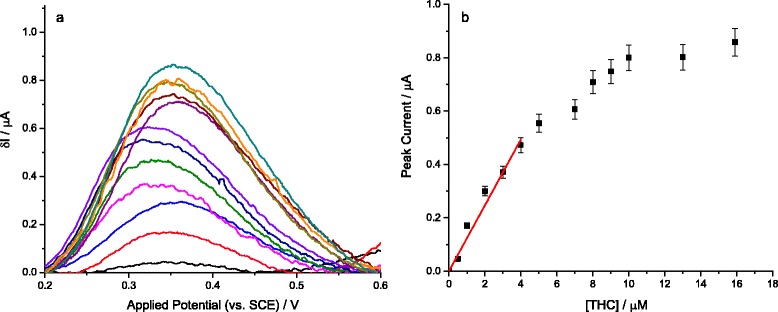
**a** Square wave voltammograms (frequency: 100 Hz, amplitude: 40 mV, step potential: 1 mV) for the oxidation of THC, seen at ca. +0.35 V (vs. SCE), on the graphite/mineral oil paste electrode. The measurement was obtained in a deoxygenated BBS (0.1 M KCl, pH = 10.0, 298 K), after immersing the electrode in identical *stationary* solutions that contained 0.50 – 16 μM THC, for 5 min. **b** The increase of the peak current with increasing THC concentration (black squares) with the correlation line through the linear range (red line, R^2^ = 0.95). The lower practical limit of detection was determined as being 0.50 μM, while the slope of the calibration curve gave a value of 0.12 μA μM^−1^ for the sensitivity of the sensor. The errors relate to separate electrode preparations

**Fig. 3:**
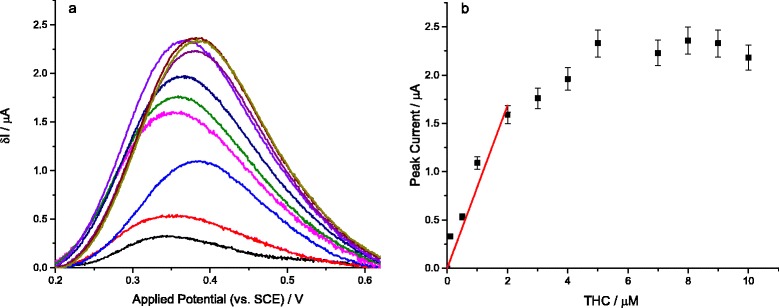
**a** Square wave voltammograms (frequency: 100 Hz, amplitude: 40 mV, step potential: 1 mV) for the oxidation of THC, seen at ca. +0.37 V (vs. SCE), on the graphite/mineral oil paste electrode. The measurement was obtained in a deoxygenated BBS (0.1 M KCl, pH = 10.0, 298 K), after immersing the electrode in identical *stirred* solutions that contained 0.10 – 16 μM THC, for 5 min. **b** The increase of the peak current with increasing THC concentration (black squares) with the correlation line through the linear range (red line, R^2^ = 0.96). The lower practical limit of detection was determined as being 0.10 μM, while the slope of the calibration curve gave a value of 0.84 μA μM^−1^ for the sensitivity of the sensor. The errors relate to separate electrode preparations

The results obtained from the stationary THC solution are presented in Fig. 2a, where peaks corresponding to the oxidation of THC, as in Scheme 1, can be observed at a peak potential of ca. +0.35 V (vs. SCE). As can be seen in Fig. 2b, the peak current shows a linear increase with increasing THC concentration up to THC concentrations of 5.0 μM; the peak height then levels off and a plateau is reached when the THC concentration is further increased. Similarly, the results obtained from the stirred THC solution can be seen in Fig. 3a, where the THC oxidation peak is seen at a peak potential of +0.37 V (vs. SCE). As previously, the peak current increases as the concentration of THC is increased, this time reaching a plateau at 4.0 μM (Fig. 3b). The errors in the calibration curves relate to separate electrode preparations.

The above results are consistent with the fact that, as more THC is present in the source solution, more THC will accumulate into the paste, resulting in higher peak currents being observed. The fact that a plateau is reached indicates that, at some point, the detection is limited by the ability of the paste to be “loaded” with more of the analyte. Comparing the THC concentration at which the plateau is reached under stationary vs. stirred conditions, the reason it is reached at lower THC concentrations when the solution is stirred is because the stirring replenishes the material that is depleted near the electrode surface, as the analyte transfers into the paste. This leads to more material accumulating into the paste at a given concentration.

Under stationary conditions, THC concentrations as low as 0.50 μM could be practically detected, while stirring the solution lowered the practical limit of detection to 0.10 μM; again this is due to the fact that stirring results in more material being present near the electrode surface and hence available to accumulate into the carbon paste. *Theoretical* limits of detection (LODs) were calculated by extrapolating the calibration curve and using the equation LOD = 3σ/*s*, where σ is the measured standard deviation of the signal in the absence of the target and s is the sensitivity of the sensor [27]. These LOD values were determined as being 0.48 nM and 0.41 nM for the stationary and stirred THC aqueous buffer solutions respectively. Limits of quantification (LOQ) were also calculated by again extrapolating the calibration curve but using the equation LOQ = 10σ/*s* [27], where the variables are the same as in the LOD equation. The calculated values were 1.61 nM and 1.38 nM for the stationary and stirred THC aqueous buffer solutions respectively. Lastly, the slope of each calibration curve gave values for the sensitivity of the sensor of 0.12 μA μM^−1^ and 0.84 μA μM^−1^ for the stationary and stirred THC aqueous buffer solutions respectively.

### Detecting THC in a stationary synthetic saliva solution

Focusing on the same THC concentration range as in Section 3.2.1, the graphite/mineral oil paste was tested in THC synthetic saliva solutions of concentrations of 0.50 – 16 μM. The same conditions and carbon paste electrode as in the Section above were used.

The graphite/mineral oil carbon paste electrode was therefore immersed in a stationary deoxygenated synthetic saliva/borate buffer solutions of pH = 10.0 that contained a known amount of THC (between 0.50 and 16 μM) and 0.1 M KCl as the supporting electrolyte. This was again done under open circuit conditions, using the same fixed 5 min pre-concentration time. Oxidative square wave voltammetric scans were then run in a deoxygenated aqueous borate buffer solution of pH 10.0, which only contained 0.1 M KCl, in the same potential range (between +0.20 V and +0.60 V [vs. SCE]) and using the same values of 100 Hz, 1 mV and 40 mV for the frequency, step potential and amplitude, respectively, as in the Section above.

**Fig. 4:**
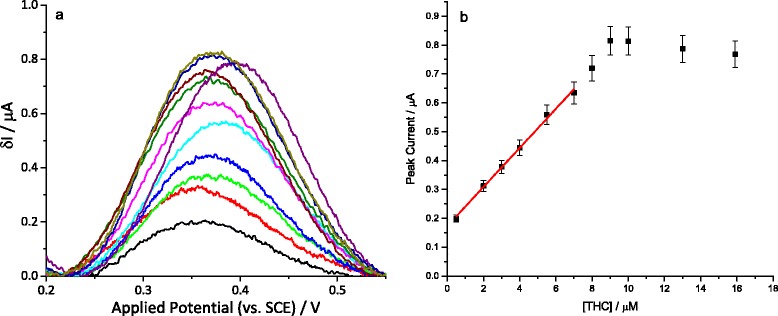
**a** Square wave voltammograms (frequency: 100 Hz, amplitude: 40 mV, step potential: 1 mV) for the oxidation of THC, seen at ca. +0.37 V (vs. SCE), on the graphite/mineral oil paste electrode. The measurement was obtained in a deoxygenated BBS (0.1 M KCl, pH=10.0, 298 K), after immersing the electrode in *stationary* deoxygenated synthetic saliva/BBS solutions that contained 0.10 – 16 μM THC, for 5 minutes. **b** The increase of the peak current with increasing THC concentration (black squares) with the correlation line through the linear range (red line, R^2^ = 0.99). The lower practical limit of detection was determined as being 0.50 μM, while the slope of the calibration curve gave a value of 0.067 μA μM^−1^ for the sensitivity of the sensor. The errors relate to separate electrode preparations

The results obtained from the stationary THC solution are presented in Fig. 4a, where peaks corresponding to the oxidation of THC, as in Scheme 1, can be observed at a peak potential of +0.37 V (vs. SCE). As can be seen in Fig. 4b, the peak current shows a linear increase with increasing THC concentration up to THC concentrations of 7.0 μM; a plateau is reached when the THC concentration is further increased. The errors in the calibration curves relate to separate electrode preparations.

The peak current increase was expected given that more and more THC will accumulate into the paste if a higher amount of THC is present in the source solution. Again as expected, the uptake is at some point limited by the paste, this resulting in the observed plateau. THC concentrations as low as 0.50 μM could be practically detected while the slope of the calibration curve gave a value of 0.067 μA μM^−1^ for the sensitivity of the sensor. Theoretical LODs and LOQs were not here calculated as the linear range was between 0.5 and 7.0 μM.

Table 1 summarises the theoretical limits of detection for THC found in literature [11–13, 26, 28–30]. As expected, given the need for sensitive detection, sensitive methodologies do exist, with limits of detection of as low as 3.2 nM having been reported [26]. However, the LOD values for stirred and unstirred buffered matrices here calculated are much lower than those tabulated. It is worth highlighting that the low *practical* limits of detection here determined represent an observable signal.

**Table 1:** Summary of limits of detection of THC found in literature

Reported LOD in [] units	Sample	Method	Reference
3.2 nM	oral samples	LC-MS	26
3.2 nM	saliva	HPLC/ED	11
4.7 nM	brain tissue	HPLC/ED	28
47 nM	urine	GC	29
120 nM	hair	HPLC/ED	30
1 micro M	saliva	ED	12
25 micro M	BBS	ED	13

Literature values for THC and THC-COOH ([28]) limits of detection

*LC* Liquid Chromatography, *MS* Mass Spectrometry, *HPLC* High Performance Liquid Chromatography, *ED* Electrochemical Detection, *GC* Gas Chromatography

## Conclusions

“Absorptive stripping voltammetry” has been applied to the detection of THC in water and saliva. An optimised carbon paste, made of graphite powder and mineral oil, was exploited in the accumulation of THC, under open circuit conditions and using a 5 min pre-concentration time.

By testing the sensor in water and synthetic saliva samples of known THC concentrations, analytical *practical* lower limits of detection (LODs) of 0.50 μM and 0.10 μM were obtained for THC in stationary and stirred aqueous borate buffer solutions, respectively, and 0.50 μM for THC in stationary synthetic saliva solutions. Theoretical LOD values of 0.48 nM and 0.41 nM were also calculated for the stationary and stirred buffer systems respectively. As Table 1 clearly demonstrates, the theoretical limits of detection here determined are much lower than those reported in the literature. Importantly, the *practical* limit of detection of 0.50 μM determined in synthetic saliva, and which corresponds to an *observed* signal, is comparable to theoretical literature values, usually *calculated*. The limit of detection of 0.50 μM is also practically useful in terms of road side detection, this also clearly illustrating the value of absorptive stripping voltammetry. This work opens up further studies into different ways of taking advantage of the properties of carbon paste electrodes [31].
